# Supplementary material for: Splice-Junction-Based Mapping of Alternative Isoforms in the Human Proteome
Source: Cell Rep. Author manuscript; Available in PMC 2020 Jan 15. (PMC6961840; doi:10.1016/j.celrep.2019.11.026)
Supplement: 2 [file NIHMS1546469-supplement-2.zip › DF1/PXD010154/Testis/Testis_52_SYCP1_VNFLPVLEQNSEGLSR.pdf]

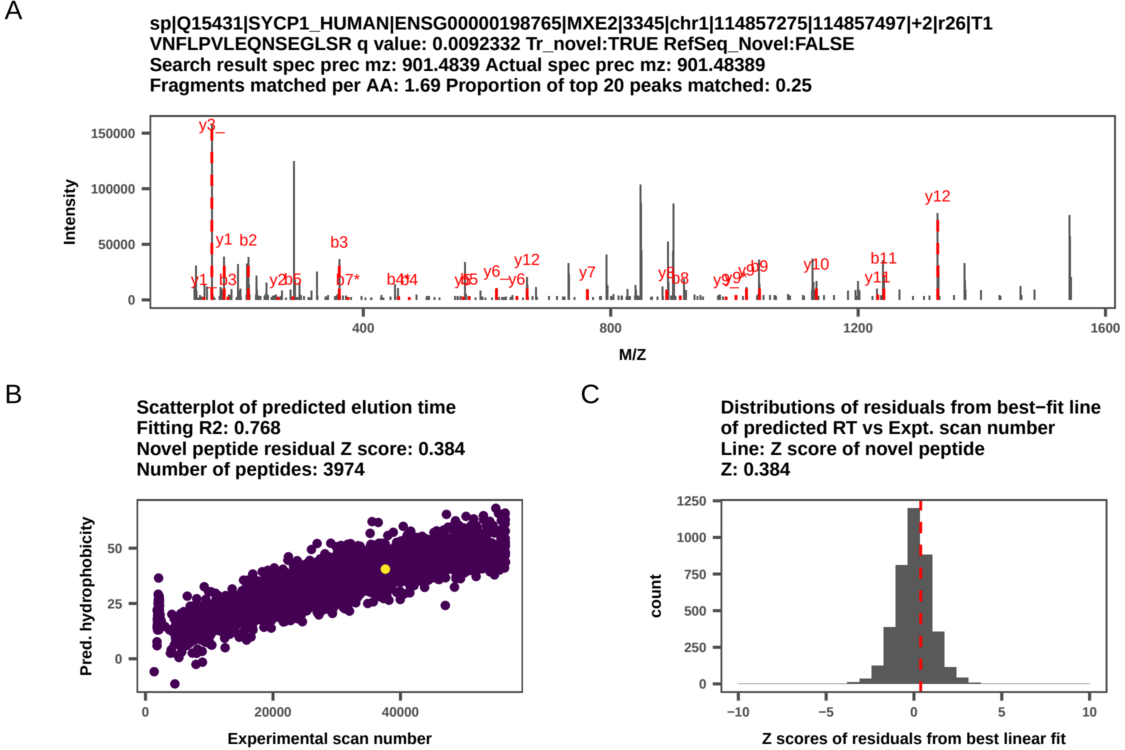
A
sp|Q15431|SYCP1_HUMAN|ENSG00000198765|MXE2|3345|chr1|114857275|114857497|+2|r26|T1
VNFLPVLEQNSEGLSR q value: 0.0092332 Tr_novel:TRUE RefSeq_Novel:FALSE
Search result spec prec mz: 901.4839 Actual spec prec mz: 901.48389
Fragments matched per AA: 1.69 Proportion of top 20 peaks matched: 0.25
Intensity
150000
100000
50000
0
y1
b2
b3
y12
y7
y10
b11
400
800
1200
1600
M/Z
B
Scatterplot of predicted elution time
Fitting R2: 0.768
Novel peptide residual Z score: 0.384
Number of peptides: 3974
Pred. hydrophobicity
50
25
0
0
20000
40000
Experimental scan number
C
Distributions of residuals from best−fit line
of predicted RT vs Expt. scan number
Line: Z score of novel peptide
Z: 0.384
count
1250
1000
750
500
250
0
−10
−5
0
5
10
Z scores of residuals from best linear fit
